# Statistical optimization of fibrinolytic enzyme production by *Pseudoalteromonas* sp. IND11 using cow dung substrate by response surface methodology

**DOI:** 10.1186/2193-1801-3-60

**Published:** 2014-01-30

**Authors:** Ponnuswamy Vijayaraghavan, Samuel Gnana Prakash Vincent

**Affiliations:** International Centre for Nanobiotechnology, Centre for Marine Science and Technology, Manonmaniam Sundaranar University, Rajakkamangalam–629 502, Kanyakumari District, Tamil Nadu India

**Keywords:** Fibrinolytic enzymes, Response surface methodology, *Pseudoalteromonas* sp. IND11, Cow dung, Solid-state fermentation

## Abstract

Fibrinolytic enzymes are agents that dissolve fibrin clots. These fibrinolytic agents have potential use to treat cardiovascular diseases, such as heart attack and stroke. In the present article, a fibrinolytic enzyme producing *Pseudoalteromonas* sp. IND11 was isolated from the fish scales and optimized for enzyme production. Cow dung was used as a substrate for the production of fibrinolytic enzyme in solid-state culture. A two-level full factorial design was used for the screening of key ingredients while further optimization was carried out using the central composite design. Statistical analysis revealed that the second-order model is significant with model *F*-value of 6.88 and *R*^2^ value of 0.860. Enzyme production was found to be high at pH 7.0, and the supplementation of 1% (w/w) maltose and 0.1% (w/w) sodium dihydrogen phosphate enhanced fibrinolytic enzyme production. The optimization of process parameters using response surface methodology resulted in a three-fold increase in the yield of fibrinolytic enzyme. This is the first report on production of fibrinolytic enzyme using cow dung substrate in solid-state fermentation.

## Introduction

Fibrin is the main component of the blood clot, and it is normally formed from fibrinogen by the action of thrombin (EC. 3. 4. 21. 5). The accumulation of fibrin in the blood vessels usually results in thrombosis. Thrombus in blood vessels or in a chamber of the heart leads to myocardial infarction and other cardiovascular diseases (CVDs). For thrombolytic therapies, both injection and oral administration of thrombolytic agents have been extensively investigated. Based upon their mechanism of activation of the fibrinolytic system, fibrinolytic agents are classified into two types. One is plasminogen activator such as tissue-type plasminogen activator (t-PA) (Collen and Lijnen 2004) and urokinase (Duffy 2002). The other one is plasmin-like fibrinolytic enzymes, which can directly degrade the fibrin in blood clots, thereby dissolving the thrombi rapidly. Examples of that type of protease are nattokinase (Sumi et al. 1987) and lumbrokinase (Mihara et al. 1991). Urokinase and t-PA are still widely used in thrombolytic therapy, but these agents have some undesirable side effects when orally administered and are expensive also (McGrath et al. 1985; Bode et al. 1996). Therefore, the search for safer thrombolytic agents from other sources is ongoing.

Microbial fibrinolytic protease is considered as a potent fibrinolytic agent to treat CVDs (Mine et al. 2005). Many thrombolytic agents have been identified and characterized from different sources (Fujita et al. 1993; Peng et al. 2003; Jeong et al. 2004; Wang et al. 2006). Although microbial fibrinolytic enzymes have been extensively studied, only few reports are available concerning statistical medium optimization (Liu et al. 2005; Deepak et al. 2010; Mahajan et al. 2012). In recent years, an attempt was made to isolate a potent fibrinolytic enzyme producing organism from marine environment (Mahajan et al. 2012). The newly established genus *Pseudoalteromonas* contains numerous species that synthesize biologically active molecules (Holmstrom and Kjelleberg 1999). The protease production of *Pseudoalteromonas* sp. has been reported by various researchers (Porro et al. 2003; Olivera et al. 2007; Xiong et al. 2007; Wang et al. 2008). Thrombolytic enzyme secreting marine bacteria *Alteromonas piscicida*was isolated by Demina et al. (1990). Hence, it is worthwhile to screen this *Pseudoalteromonas* sp. for fibrinolytic enzyme secretion and statistical optimization for enzyme production.

In the present article, solid-state fermentation (SSF) was employed for the production of fibrinolytic enzyme using cow dung substrate. Cattle manure consists of cellulose (35.4%), hemicelluloses (32.6%), ash (13.3 - 13.4%) and nitrogen (1.2 – 1.6%) (Misra et al. 2003). Cow dung substrate was effectively used for the production of alkaline protease recently in SSF (Vijayaraghavan and Vincent 2012; Vijayaraghavan et al. 2012). In enzyme bioprocess, designing a suitable medium for maximum production is of critically important because the medium components significantly affect the product yield. Considering its cheap cost and availability, an attempt was made to use cow dung as a substrate for the production of fibrinolytic enzyme from *Pseudoalteromonas* sp. IND11. The traditional one-at-a-time optimization strategy is simple and easy, but it fails frequently. Statistical experimental design provides an efficient approach to optimize the medium components. Fractional factorial design (FFD) is especially suitable to account for the interactions and identify the most significant components in the medium formula. A combination of factors generating a certain optimum response can be identified through factorial design and the use of response surface methodology (RSM). The statistical method is more satisfactory and effective than other classical one-at-a-time optimization strategies because it can study many variables simultaneously with a lower number of observations, thus saving time and cost (Montogomery 2001). RSM is a well-known method applied in the optimization of medium constituents and other critical variables responsible for the production of biomolecules (Hong et al. 2004) and enzymes (Long et al. 2009; Zhang et al. 2012; Farid et al. 2013).

The aim of this work was to optimize the fermentation medium by statistical approach to increase fibrinolytic enzyme production by *Pseudoalteromonas* sp. using cow dung, which is a low cost substrate.

## Methods

### Screening and identification of a fibrinolytic enzyme producing *Pseudoalteromonas* sp. IND11

*Pseudoalteromonas* sp. IND11 producing a fibrinolytic enzyme was isolated along with other bacteria from the fish scales in Kanyakumari, India. Primary screening was carried out using skimmed milk agar plates. The sample was homogenized and platted on skimmed milk agar plates (peptone, 5 g/L; beef extract, 1.5 g/L; yeast extract, 1.5 g/L; sodium chloride, 5 g/L; agar, 15 g/L; skim milk, 10 g/L). All plates were incubated at 37°C for 24 h, and a total of nine positive isolates were obtained based on their morphological and cultural characteristics. All nine organisms were cultured in nutrient broth medium (peptone, 5 g/L; beef extract, 1.5 g/L; yeast extract, 1.5 g/L; sodium chloride, 5 g/L; and casein, 10 g/L) and incubated at 37°C in a shaker (150 rpm) for 48 h. These isolates were further screened using fibrin plates. The cell-free extracts (15 μl) were dropped into the well of fibrin plate (pH 7.4). The single strain showing the largest halo zone on fibrin-agarose plate was selected and further identified. Various biochemical tests of the isolate *Pseudoalteromonas* sp. IND11 were done to identify it according to the “Bergey’s Manual of Systematic Bacteriology” (Sneath 1986) and by 16S rDNA sequencing. The genomic DNA of the isolate was extracted using genomic DNA extraction kit (QIAGEN, Germany) per manufacturer’s instructions. Amplification of the 16S rDNA was performed with universal primers 27 F (5′-AGAGTTTGATCMTGGCTAG-3′) and 1492R (5′-ACGGGCGGTGTGTRC-3′), using a research gradient Peltier Thermal cycler machine PTC-225 (USA). PCR products were isolated from the agarose gel using a QIA quick gel extraction kit (QIAGEN, Germany) and sequenced. A sequence similarity search was performed using BLAST in the NCBI database. The 612 bp 16S rDNA sequence of strain has been submitted to Genbank database and assigned accession number KF683956.

### Assay of fibrinolytic activity

Fibrinolytic activity was measured by the hydrolysis of fibrin according to the method described by Ansen (1939) with some modifications. The incubation mixture contained 2.5 ml of 1.2% fibrin (w/v), 2.5 ml of 0.1 M Tris-HCl (0.01 M CaCl_2_, pH 7.8), and a suitable amount of enzyme. The incubation was carried out at 37°C for 30 min, and the reaction was stopped by adding 5 ml of 0.11 M trichloroacetic acid containing 0.22 M sodium acetate and 0.33 M acetic acid. The absorbance of the supernatant was measured at 275 nm. A fibrinolytic unit was defined as the amount of enzyme that gave an increase in absorbency at 275 nm equivalent to 1 μg of tyrosine per minute at 37°C. The total protein content determination was performed by Lowry et al. (1951).

### Optimization for fibrinolytic enzyme production by one-variable-at-a-time approach

Cow dung was collected locally and dried for one week (sun drying). It was powdered using a mixer grinder, sieved and stored at room temperature for further use. Cow dung was used as the substrate. SSF was carried out in a 100 ml Erlenmeyer conical flask containing 2 g substrate. In the present article, the requirement of medium components including various carbon (1% [w/w], glucose, sucrose, maltose, xylose, trehalose, and starch) and nitrogen sources (1% [w/w], yeast extract, urea, casein, gelatin, beef extract, and peptone) and inorganic salts (1% [w/w], ammonium chloride, ferrous sulfate, disodium hydrogen phosphate, calcium chloride, sodium nitrate, sodium dihydrogen phosphate, and ammonium sulfate) were optimized. The physical factors such as fermentation period (24–96 h), pH (5–10), moisture (80%–160%), and inoculum (3%–15%) were also evaluated before being subjected to statistical optimization. Cow dung substrate was moistened with tris buffer (pH 8, 0.1 M) at 100% level and was treated as the control. The contents were sterilized and inoculated with 0.2 ml of 18 h grown culture broth (0.983 OD at 600 nm) under sterile conditions. The enzyme was extracted from cow dung using 20 ml double distilled water by shaking on a rotary shaker (150 rpm) for 30 min. This was centrifuged at 10000 rpm for 10 min at 4°C and the clear supernatant was used as the crude enzyme.

### Evaluation of significant components with two-level full factorial design

A two-level full factorial design (2^5^) was employed to find the key ingredients that affect fibrinolytic enzyme production. The important physical parameters (pH and moisture) and nutrient factors (maltose, casein, and sodium dihydrogen phosphate) were evaluated by statistical method. The other factors such as fermentation period and inoculum were kept at optimum level. Based on two-level full factorial design, each factor was examined at two levels (−1 for low level and +1 for high level). The variables and the levels were described in Table 1. Two-level full factorial designs were based on the following first-order polynomial model:

where *Y* is the response (fibrinolytic activity); *α*_*ij*,_*α*_*ijk*,_*α*_*ijkl*_, and *α*_*ijklm*_ were the *ij*th, *ijk*th, *ijkl*th, and *ijklm*th interaction coefficients, respectively; *α*_i_ was the *i*th linear coefficient; and *α*_0_ was an intercept.

**Table 1:** **Variables selected for 2-level full factorial design**

		Coded levels	
Factors	Units		
		−1	+1
A-pH		8	.10
B-Moisture	%	80	120
C-Maltose	%	0.1	1.0
D-Casein	%	0.1	0.5
E-NaH_2_PO_4_	%	0.05	0.25

Fibrinolytic enzyme assay was carried out in duplicates, and the average of these experimental values was taken as response *Y* (Table 2). The statistical software “Design-Expert 8.0” (StatEase Inc., Minneapolis, USA) was used to analyze the experimental results. The factors that affect fibrinolytic enzyme production significantly (*p* < 0.05) were further optimized by central composite design (CCD) and RSM.

**Table 2:** **Experimental design and results of the two level (** **2** ^**5**^ **) full factorial design**

Run	pH	Moisture	Maltose	Casein	NaH_2_PO_4_	Enzyme activity (U/ml)
1	1	−1	1	1	1	151
2	−1	−1	−1	−1	−1	638
3	1	1	−1	1	−1	274
4	−1	1	−1	1	1	1388
5	−1	1	−1	−1	−1	1118
6	1	−1	−1	−1	1	521
7	1	−1	−1	−1	−1	398
8	−1	1	1	−1	−1	844
9	−1	1	−1	1	−1	404
10	−1	1	−1	−1	1	1029
11	1	1	1	−1	−1	638
12	−1	−1	1	−1	1	631
13	1	1	1	1	−1	645
14	−1	−1	−1	−1	1	617
15	1	1	−1	1	−1	899
16	−1	−1	1	1	−1	576
17	1	−1	−1	1	−1	761
18	−1	−1	−1	1	1	652
19	1	−1	−1	1	−1	727
20	1	1	1	−1	1	384
21	1	−1	−1	1	1	684
22	−1	1	1	−1	1	727
23	−1	1	1	1	−1	576
24	−1	1	1	1	1	912
25	1	1	−1	−1	1	713
26	1	−1	1	−1	1	350
27	1	1	−1	1	1	363
28	−1	−1	1	1	1	1160
29	1	−1	1	1	−1	1251
30	1	1	1	1	1	89
31	−1	−1	1	−1	−1	748
32	1	−1	1	−1	−1	562

### Statistical optimization of fibrinolytic enzyme production in SSF by CCD and RSM

CCD was employed to optimize the fermentation conditions, namely, pH, maltose, and sodium dihydrogen phosphate at five levels (−*α*, −1, 0, +1, +*α*) (Table 3). The CCD contains a total of 20 experimental runs (8 factorial, 6 central, and 6 axial points). The experiments were conducted in duplicates, and the mean value (U/ml) of fibrinolytic activity was taken as the response (*Y*) (Table 4). The statistical software “Design-Expert 8.0” (StatEase) was used to analyze the experimental results. The experimental results of CCD were fitted with a second-order polynomial equation as shown below:

**Table 3:** **The independent variables and their levels for the central composite experimental design**

Variables	Symbol	Units		Coded levels			
			-α	−1	0	+1	+α
pH	A		6.32	7	8	9	9.68
Maltose	B	%	−0.01	0.25	0.5	1.0	1.26
NaH_2_PO_4_	C	%	−0.02	0.01	0.06	0.1	0.13

**Table 4:** **Experimental design and results of CCD**

Run	pH	Maltose	NaH_2_PO_4_	Enzyme activity (U/ml)
1	0	0	0	1028
2	0	0	0	1276
3	1	−1	−1	910
4	1.682	0	0	558
5	0	−1.681	0	683
6	0	0	0	1000
7	0	0	1.682	699
8	0	0	0	1173
9	−1	−1	−1	703
10	−1	−1	1	1186
11	1	−1	1	531
12	0	0	0	1104
13	−1	1	−1	1573
14	1	1	−1	765
15	1	1	1	462
16	0	0	−1.682	897
17	−1	1	−1	966
18	0	0	0	1055
19	−1.682	0	0	903
20	0	1.682	0	1345

For a three-factor system, the second-order polynomial equation is as follows:

where *Y* is the response; *β*_0_ is the offset term; and *β*_*i*_, *β*_*ii*_, and *β*_*ij*_ were the coefficients of linear terms, square terms, and coefficients of interactive terms, respectively. *X*_*i*_’s were A, B, and C; *X*_*ij*_’s were AB, AC, and BC (A-coded value of pH; B-coded value of maltose; C-coded value of sodium dihydrogen phosphate).

### Validation of the experimental model

To validate the model equation, experiments were conducted in triplicates for fibrinolytic enzyme production under optimum conditions predicted by the model.

## Results and discussion

### Novel compounds from the marine microbes

Marine organisms known to produce a diverse spectrum of novel metabolites are an untapped source for the discovery of new bioactive compounds (Mahajan et al. 2012). Further, it is believed that sea water, which is saline in nature and chemically closer to the human blood plasma, could provide biomolecules, in particular enzymes that could have lower or no toxicity or side effects when used for therapeutic applications (Sabu 2003). Recently many researchers had focused their efforts on isolating and screening of microorganisms for enzyme production with high fibrinolytic activity from various sources (Jeong et al. 2001; Lee et al. 2001; Agrebi et al. 2009). Although fibrinolytic enzymes have been isolated from various organisms, the quest for new fibrinolytic enzymes has not been stopped yet (Mahajan et al. 2012). In this article, a marine isolate *Pseudoalteromonas* sp. IND11 was subjected to screening and optimizing for fibrinolytic enzyme production. Many organisms from the genus *Pesudoalteromonas* secreted proteolytic enzymes, for example, *Pseudoalteromonas* sp. strain CP76 (Porro et al. 2003) and *Pseudoalteromonas* sp. NJ276 (Wang et al. 2008).

### Isolation, screening, and identification of fibrinolytic enzyme producing *Pseudoalteromonas* sp. IND11

**Figure 1:**
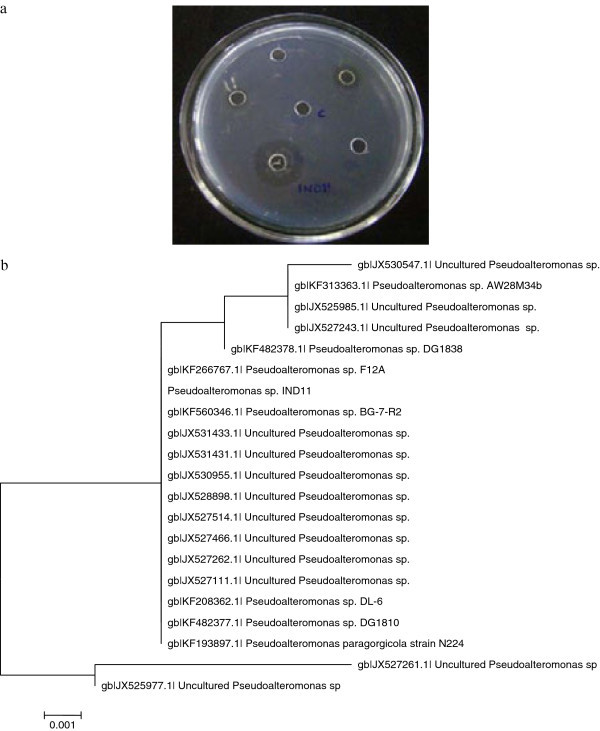
**Analysis of fibrinolysis on fibrin-agarose plate (a).** Phylogenetic relationships of strain IND11 and other closely related *Pseudoalteromonas* sp. **(b)**.

In the present article, nine fibrinolytic enzymes secreting organisms were isolated from the fish scales, obtained from Arabian sea, 25 km from Kanyakumari coast, Tamilnadu, India. The extracellular fibrinolytic enzyme production was determined by fibrin plate method. Among the isolates, *Pseudoalteromonas* sp. IND11 showed more activity; hence this was selected for further studies (Figure 1a). It was Gram - negative, rod - shaped and positive in oxidase test. It showed positivity in tests of catalase and Voges - Proskauer and in casein -, and starch - hydrolysis. The strain was negative in tests of nitrate - reduction, urease -, indole -, gas-production and citrate utilization. The isolate was identified as *Pseudoalteromonas* sp. The 612 bp 16S rDNA sequence of *Pseudoalteromonas* sp. IND11 has been submitted to the GenBank database and assigned accession number KF683956. A phylogenetic tree was derived from 16S rDNA sequences, and it showed the phylogenetic position of *Pseudoalteromonas* sp. IND11 to closely related species in the genus *Pseudoalteromonas* (Figure 1b).

### Cow dung: a cheap substrate for enzyme production

This article has showed that cow dung can be used as a solid substrate for fibrinolytic enzyme production. The selection of an ideal substrate for the production of any metabolite is an important factor from an industrial point of view. An ideal substrate should be available throughout the year (Pandey et al. 2000). Reports on SSF of cow dung substrate for the production of fibrinolytic enzyme are limited or, perhaps, not available. Recently, we used cow dung substrate for the production of proteolytic enzymes (Vijayaraghavan and Vincent 2012; Vijayaraghavan et al. 2012), and further now we use the cow dung substarte for fibrinolytic enzyme production. Based on the results obtained from the present article, cow dung is an ideal substrate for fibrinolytic enzyme production. Cow dung substrate supplies nutrients to the microbial culture and anchorage for the growing cells. This is the first report on production of fibrinolytic enzyme from a marine bacterial isolate, *Pseudoalteromonas* sp. IND11, using statistical experimental design and RSM in optimization of its production under SSF.

### Preliminary screening of nutrients and physical factors for statistical optimization

**Figure 2:**
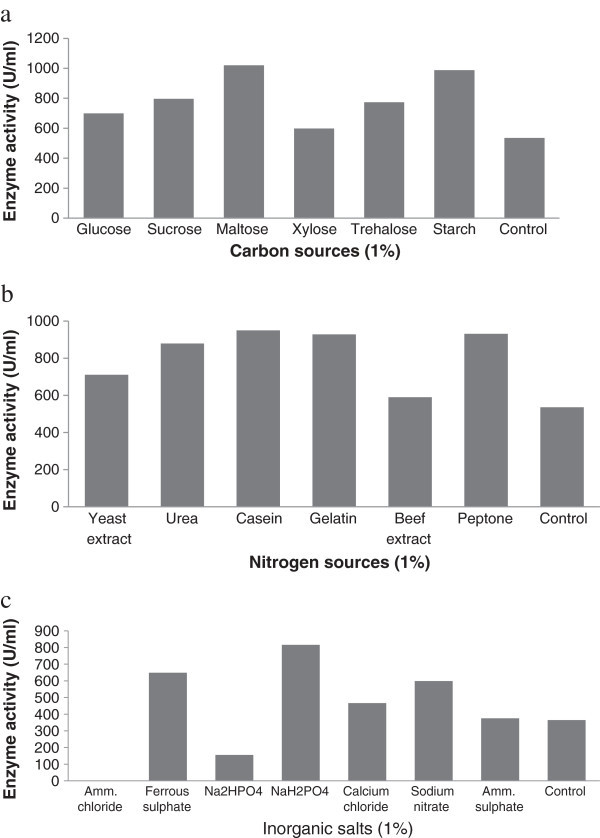
**Effect of various carbon (a), nitrogen (b) and inorganic salts (c) on production of fibrinolytic enzymes.**

The microbial strain is unique in their molecular, biochemical, metabolic, and enzyme production properties. Hence, an in-depth knowledge of kinetics and catalytic behavior during protease production from any new strain is a prerequisite for the evaluation of its biotechnological potential (Prakasham et al. 2006). The nutrient factors and physical parameters were optimized to increase fibrinolytic enzyme secretion. The effect of carbon sources was shown in Figure 2a. In this article, among all the supplementary carbon sources (1% [w/w]), maltose has been found to be the best source for fibrinolytic enzyme production. These results were in accordance with reported protease production in the presence of different sugars (Ellaiah et al. 2002). When different concentrations of maltose were added, maltose (1% [w/w]) supported the maximum fibrinolytic enzyme production (1038 U/ml). It was shown in bacteria that the production of fibrinolytic enzyme was induced by maltose in the culture medium (Liu et al. 2005). The other sources such as glucose, sucrose, xylose, trehalose, and starch also supported fibrinolytic enzyme production. Among nitrogen sources, the addition of casein supported maximum fibrinolytic enzyme production (Figure 2b). When different concentrations of casein were supplemented, casein at 1.5% supported the maximum fibrinolytic enzyme production (1006 U/ml). The supplemented casein stimulated fibrinolytic enzyme production. These results are in accordance with the observation made with *Bacillus* sp. strain AS-S20-1 (Mukherjee and Rai 2011). The sources such as yeast extract, urea, gelatin, beef extract, and peptone also enhanced fibrinolytic enzyme production. In SSF, the addition of inorganic salts enhanced fibrinolytic enzyme production except ammonium chloride. Among the inorganic salts, sodium dihydrogen phosphate enhanced fibrinolytic enzyme production. These results are in accordance with the observation made with *Bacillus subtilis* (Artemov and Samuilov 1990). This is because upon translocation across the plasma membrane, serine protease is bound to the outer surface of cell membranes. This bounded protease inhibits extracellular protease synthesis and the metal ions induce the displacement of this enzyme. This could be the possible reason for showing major effect of Na^+^ ion on fibrinolytic enzyme production (Mahajan et al. 2012). Ammonium chloride (1% [w/w]) totally inhibited fibrinolytic enzyme production (Figure 2c). The repression of the synthesis of protease by excess ammonium has been reported earlier (Kole et al. 1988).

In SSF, moisture content is one of the critical factors for fibrinolytic enzyme production. One-factor-at-a-time experiment revealed that moisture (140%) significantly affected enzyme production. Among the several factors that are important for microbial growth and enzyme production under SSF, moisture content is a critical factor (Pandey et al. 2000). Alkaline protease production by microbial strains strongly depends on the extracellular pH (Ellaiah et al. 2002). In the present article, the pH of the substrate greatly affected enzyme production. Hence, these variables were also selected for statistical optimization experiments.

### Two-level full factorial experimental design and an analysis of main effects

The most significant factors such as pH, moisture, maltose, casein, and sodium dihydrogen phosphate were selected for optimization studies. The matrix developed by the two-level full factorial design and the results were shown in Table 2. The fibrinolytic enzyme production varied from 89 to 1388 U/ml. According to the two-level full factorial design, the optimum medium compositions were as follows: pH 8.0, 120% moisture, 0.1% maltose, 0.5% casein, and 0.25% sodium dihydrogen phosphate. The analysis of variance (ANOVA) was used to analyze the main effects and was shown in Table 5. The model *F*-value of 96.26 implies that the model is significant. There is only a 0.16% chance that a “Model *F*-value” this large could occur due to noise. Values of “Prob > *F*” less than 0.05 indicate that model terms are significant. In this model, enzyme production was significantly affected by pH (<0.01), maltose (<0.05), and sodium dihydrogen phosphate (<0.05). The coefficient estimate was negative to pH, maltose and sodium dihydrogen phosphate. This states that the lower levels of pH and maltose and sodium dihydrogen phosphate concentrations would benefit fibrinolytic enzyme production. The coefficient estimate was positive to moisture and casein; these indicated that the increase of moisture content and casein concentration in the cow dung medium will support for enzyme production. The “predicted *R*-squared” of 0.865 is in reasonable agreement with the “adjusted *R*-squared” of 0.987. Adequate precision measures the signal-to-noise ratio. A ratio greater than 4 is desirable. In this model, the ratio of 43.54 indicates an adequate signal. This model can be used to navigate the design space. Neglecting the insignificant variables, the model equation for fibrinolytic enzyme production is as follows:

**Table 5:** **ANOVA table for two**-**level full factorial design**

Source	Sum of squares	df	Mean square	F- Value	p-value
Model	3.777E + 006	28	1.349E + 005	90.26	0.0016
pH	5.161E + 005	1	5.161E + 005	345.36	0.0003
Moisture	10368	1	10368	6.94	0.0781
Maltose	27730	1	27730	18.54	0.023
Casein	4900	1	4900	3.28	0.167
NaH_2_PO_4_	14792	1	14792	9.90	0.05
Residual	4483.37	3	1493		
Cor total	3.781E + 006	31			

Final equation in terms of coded factor:

The three significant factors such as pH, maltose, and casein were further optimized with CCD.

### Response surface methodology

The effect of the three variables (pH, maltose, and sodium dihydrogen phosphate) on fibrinolytic enzyme production was evaluated by CCD and RSM. The CCD model helps to study the interactions between the various variables, and RSM helps to explore the optimum concentrations of each of the variables. The maximum activity of the fibrinolytic enzyme was observed at run 13 (Table 4). The results obtained from CCD were analyzed using ANOVA. The model *F*-value of 6.88 implies that the model is significant (Table 6). There is only a 0.29% chance that a “Model *F*-value” this large could occur due to noise. Values of “Prob > *F*” less than 0.05 indicate that model terms are significant. In this case, A, B, AC, A^2^, and C^2^ are significant model terms. The second-order polynomial model was used to correlate the independent variables with fibrinolytic enzyme activity. The coefficient of determination (*R*^2^) was calculated to be 0.86, indicating that the model could explain 86% of the variability. A value of >0.75 indicates appropriate for the model. The “Lack-of-Fit *F*-value” of 3.22 implies that the lack of fit is not significant relative to the pure error. There is only an 11.27% chance that a “Lack-of-Fit *F*-value” this large could occur due to noise. “Adequate precision” measures the signal-to-noise ratio. A ratio greater than 4 is desirable. A ratio of 10.02 indicates the adequate signal. The response (*Y*) was well fitted with a quadratic second-order polynomial equation.

**Table 6:** **ANOVA for response surface quadratic model**

Source	Sum of squares	df	Mean square	***F*** Value	***p***-value	
Model	1.392E + 006	9	1.546E + 005	6.88	0.0029	Significant
A-pH	4.010E + 005	1	4.010E + 005	17.83	0.0018	
B-Maltose	1.758E + 005	1	1.758E + 005	7.82	0.0189	
C-NaH_2_PO_4_	44.14	1	44.14	1.963E-003	0.9655	
AB	93312	1	93312	4.15	0.069	
AC	3.925E + 005	1	3.925E + 005	17.46	0.0019	
BC	5000	1	5000	0.22	0.6474	
A^2^	2.075E + 005	1	2.075E + 005	9.23	0.0125	
B^2^	5628.9	1	5628.9	0.25	0.6277	
C^2^	1.483E + 005	1	1.483E + 005	6.59	0.0280	
Residual	2.249E + 005	10	22485.08			
Lack of fit	1.715E + 005	5	34307.37	3.22	0.1127	Not significant
Pure error	53314	5	10662.80			
Cor total	1.617E + 006	19				

Final equation in terms of coded factors:

where A is the pH of the substrate, B is maltose (%), and C is sodium dihydrogen phosphate (%).

**Figure 3:**
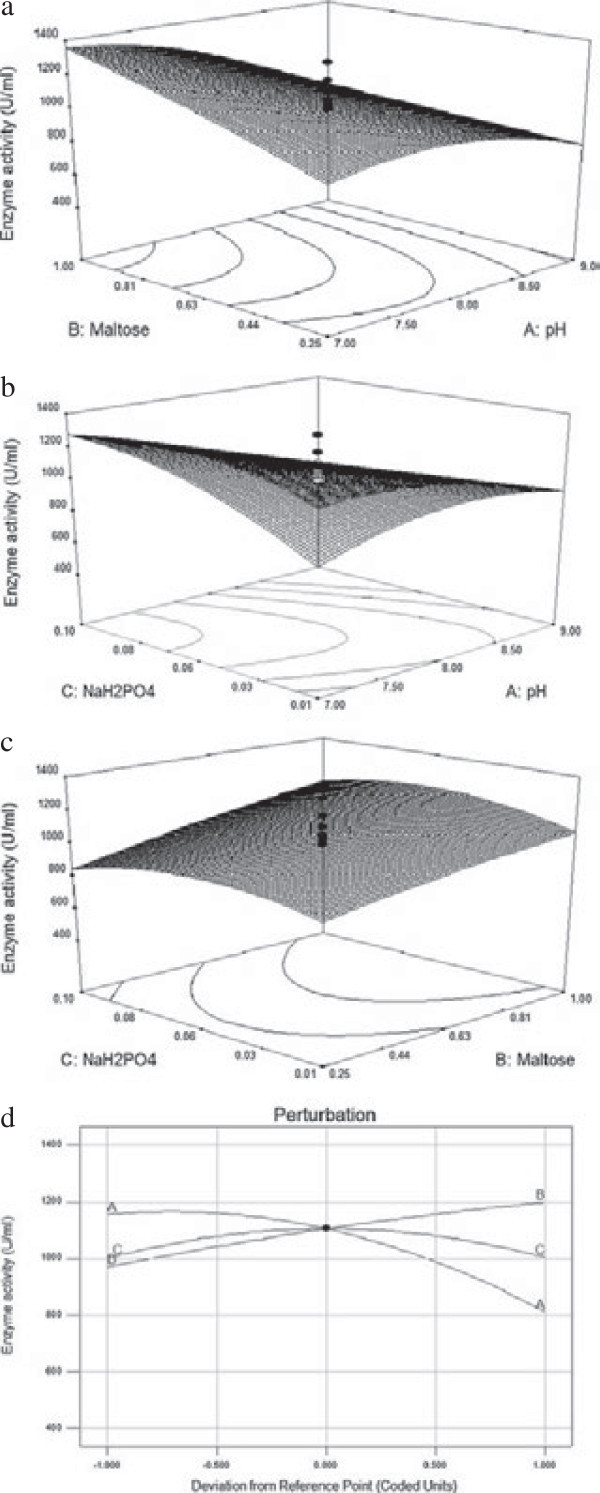
**Three dimensional response surface curves showing the effect of interactions of (a) pH and maltose,** **(b) pH and NaH**_**2**_ **PO**_**4**_ **(c) Maltose and NaH**_**2**_ **PO**_**4**_ **(d) Perturbation graph shows the effect of pH (a), maltose (b) and NaH**_**2**_ **PO**_**4**_ **.**

The three-dimensional surface plots show the combined effect of two independent variables for fibrinolytic enzyme production, while the third variable was kept at zero-coded level (Figure 3a–c). A perturbation plot compared the effect of the entire factor at a particular point in the design space (Figure 3d). Among the variables used for RSM, medium pH had a significant effect on fibrinolytic enzyme production compared with other variables. The perturbation graph shows that the factor maltose had a significant role on fibrinolytic enzyme production. Before optimization, the enzyme production was 505 U/ml, and threefold increased enzyme production was achieved after optimizing the medium by RSM. The predicted maximum fibrinolytic enzyme production was estimated to be 1573 U/ml. To validate the experimental design, experiments were conducted in triplicates in optimized conditions, and a fibrinolytic activity of 1610 U/ml was obtained. This experimental value (1610 U/ml) is in good agreement with that of the predicted value that validates the model design.

Recently, Al- Nahas et al. (2011) found the fibrinolytic potential of exopolysaccharide synthesized by *Pseudoalteromonas* spp. from the deep sea sediments. *Pseudoalteromonas* sp. synthesizes variety of active compounds (Holmstrom and Kjelleberg 1999). The isolate *Pseudoalteromonas* sp. IND11 grows on cow dung substrate, so it is worthwhile to use cow dung substrate for the production of other useful compounds too. Although fibrinolytic enzyme producing organisms from various marine isolates were reported, only very few reports are available on the secretion of fibrinolytic enzyme by *Pseudoalteromonas* sp. These kinds of study not only exploit this organism for the optimized production of fibrinolytic enzymes but also help to synthsize other useful compounds.

### Optimization and validation

Validation of the predicted results was done under optimized conditions in three independent experiments. In this model, the experimental fibrinolytic activity of 1365 U/ml was obtained which correlated to the predicted activity (1340 U/ml) confirming the rationality of the model. This is 3 fold higher than that obtained before optimization. Thus, overall 3 fold increase in fibrinolytic activity was observed after optimization.

## Conclusion

A fibrinolytic enzyme-secreting *Pseudoalteromonas* sp. IND11 was grown on cow dung substrate in solid-state culture. The fermentation medium for fibrinolytic enzyme production was optimized using two-level full factorial design and CCD. In the present article, supplementation of 1% (w/w) maltose and 0.1% (w/w) sodium dihydrogen phosphate increased fibrinolytic enzyme production. The optimized medium showed 1573 U/ml of fibrinolytic activity, which is three times higher than the unoptimized medium.
